# Evaluating the Efficacy and Safety of *Limnospira platensis* Phycobiliproteins as a Functional Feed Additive for Oxidative Status Modulation in the Pacific Oyster

**DOI:** 10.3390/antiox15091067

**Published:** 2026-08-26

**Authors:** Olga Gostyukhina, Elina Chelebieva, Tatyana Kukhareva, Maria Podolskaya, Vitaliy Parfenov, Andrey Borovkov, Aleksandra Andreyeva

**Affiliations:** 1 Laboratory of Ecological Immunology of Aquatic Organisms, A.O. Kovalevsky Institute of Biology of the Southern Seas of RAS, 119991 Moscow, Russia; gostyukhina.olga@ibss-ras.ru (O.G.); e.chelebieva@ibss-ras.ru (E.C.); kukhareva@ibss-ras.ru (T.K.); vitaliy.par@ibss-ras.ru (V.P.); lab_eimg@ibss-ras.ru (A.A.); 2 Department of Biotechnology and Phytoresources, A.O. Kovalevsky Institute of Biology of the Southern Seas of RAS, 119991 Moscow, Russia; biotex@ibss-ras.ru

**Keywords:** Pacific oyster, phycobiliproteins, lipid peroxidation, protein oxidative modification, antioxidant activity, aquaculture

## Abstract

Despite the recognized antioxidant properties of phycobiliproteins (PBPs) from *Limnospira platensis*, their potential application in bivalve aquaculture—particularly regarding their modulation of endogenous antioxidant defenses—remains largely unexplored. This study evaluated the effects of an aqueous PBP extract at concentrations of 2, 20, and 80 µg/mL on antioxidant parameters in the Pacific oyster *Magallana gigas* following 24, 48 and 96 h exposure periods. Activities of superoxide dismutase (SOD), catalase (CAT), and glutathione peroxidase (GPx), levels of lipid peroxidation (TBARS) and protein oxidation, and expression of corresponding genes in gills and hepatopancreas have been evaluated. PBPs elicited significant effects on prooxidant–antioxidant balance in oyster tissues. Low concentrations (2–20 µg/mL) enhanced SOD and CAT activities and reduced protein oxidation, indicating protective effects. The highest concentration (80 µg/mL) suppressed enzyme activities—particularly GPx—while elevating TBARS, suggesting prooxidant effects at excessive doses. Tissue-specific responses were observed, with hepatopancreas more vulnerable to oxidative stress than gills. Notably, GPx transcription was upregulated at 80 µg/mL of PBP extract despite suppressed activity, implying its post-transcriptional regulatory properties. These findings demonstrate that *L. platensis* PBPs modulate oyster antioxidant status in a biphasic manner, with moderate doses conferring stimulation and high doses inducing oxidative stress. This study provides the first integrative evidence of PBP effects on enzymatic, and transcriptional antioxidant defenses in a commercially important bivalve, underscoring their promise as functional bioactive additives while emphasizing the need for careful dose optimization.

## 1. Introduction

Oxidative stress and its biological implications have drawn considerable research interest, owing to its rising prevalence across vertebrate and invertebrate species exposed to diverse environmental stressors [1]. This has prompted extensive investigation into oxidative stress-related pathologies, ultimately leading to the development of numerous antioxidant agents. Antioxidants serve to mitigate the deleterious effects of oxidative stress, thereby contributing to cytoprotection and organismal resilience. While both natural and synthetic antioxidants are available, natural compounds have gained prominence due to their efficacy and favorable safety profiles, typically associated with minimal or no adverse effects [2,3].

Cyanobacteria are recognized as emerging sources of bioactive compounds, including fatty acids, carotenoids, polysaccharides, and pigment proteins [4,5,6]. Among these, pigment proteins have attracted particular interest due to their potential biomedical applications [7]. Cyanobacteria are notably rich in phycobiliproteins (PBPs), which can constitute approximately 60% of total protein and up to 20% of cellular dry weight [8]. These pigments function as photosynthetic antenna complexes in cyanobacteria, red algae, and cryptophytes, efficiently harvesting light energy and transferring it to chlorophyll during photosynthesis [8]. Owing to their antioxidant capacity, PBPs have also been investigated as potential pharmacological and medicinal agents [9]. Spirulina (taxonomically referred to as *Limnospira platensis*) is a non-toxic cyanobacterium commonly found in tropical and subtropical lakes with high pH and elevated carbonate/bicarbonate concentrations [10]. Beyond its substantial protein content, *L. platensis* provides vitamins, minerals, unsaturated fatty acids, and other nutrients, making it a balanced natural food product widely used as a dietary supplement [11]. In *L. platensis*, the primary PBPs are C-phycocyanin (C-PC), the major pigment, and allophycocyanin (APC), present in considerably smaller amounts [12].

Over recent decades, PBPs have been extensively investigated for their antioxidant and free radical-scavenging properties using various in vitro and in vivo experimental models [12]. Given their proven bioactivity and safety as dietary components, PBPs have attracted growing interest as functional additives in animal nutrition. This is particularly relevant in aquaculture, where nutrition is a critical determinant of production success, directly influencing growth performance, survival, and overall health of cultured organisms. Microalgae are widely employed in this sector as live feeds, dietary supplements, bioremediating agents for water quality management, growth promoters, and natural pigmentation enhancers [13]. Despite their established nutritional and bioactive value, however, research specifically addressing the antioxidant effects of PBPs in aquaculture remains limited. Nonetheless, several studies have indicated that phycocyanin derived from blue-green algae holds promise as a functional food ingredient [14,15,16]. As a natural source of PBPs and other biologically active compounds, *L. platensis* has long been utilized as a dietary supplement in animal nutrition [17]. More recently, there has been growing interest in its application as a feed additive in the farming of fish, shrimp, and ornamental species [13].

The antioxidant potential of *L. platensis*-supplemented diets in fish aquaculture has been corroborated by several experimental studies. In rainbow trout (*Oncorhynchus mykiss*), long-term (10-week) dietary spirulina supplementation reduced hepatic and plasma lipid peroxidation while significantly upregulating hepatic gene expression of superoxide dismutase (SOD) and catalase (CAT) [18]. Similarly, dietary *L. platensis* enhanced glutathione reductase and CAT activities and decreased malondialdehyde (MDA) formation in the muscle of Nile tilapia (*Oreochromis niloticus*) [19]. Despite these encouraging outcomes in finfish, the role of PBPs and *L. platensis* derivatives in the health management of invertebrate aquaculture species remains considerably underexplored. To date, studies specifically investigating the antioxidant effects of functional diets in invertebrates remain scarce. For instance, an eight-week dietary supplementation with 6–8 g/kg of spirulina powder in Pacific white shrimp (*Litopenaeus vannamei*) resulted in significantly reduced MDA levels (*p* ≤ 0.05), alongside increased activities of hemolymph glutathione peroxidase (GPx), SOD, and CAT [20]. In bivalve aquaculture, however, the integration of functional algal feed additives into diets for larvae, spat, or broodstock in hatcheries is still limited, despite microalgae constituting the primary food source for these organisms.

Hatcheries are poised to play a pivotal role in the global expansion of oyster aquaculture by enabling the production of spat independently of natural spawning cycles. Prior to spawning, adult broodstock are typically removed from the environment and maintained indoors for up to three months under controlled conditions of pH, temperature, salinity, and photoperiod [21,22]. Under these managed conditions, broodstock require a balanced diet to sustain their health status and ensure high-quality spawning. In this context, supplementing standard diets with functional bioactive additives represents a promising strategy for advancing oyster aquaculture.

Nevertheless, fundamental knowledge concerning the effects of *L. platensis* and its bioactive derivatives on the antioxidant status of commercially important bivalve species—such as the Pacific oyster (*Magallana gigas*)—is still lacking. Therefore, the primary objectives of the present study were to investigate the effects of an aqueous extract of PBPs derived from *L. platensis* on: (i) the activity of key antioxidant enzymes (SOD, CAT, and GPx), (ii) lipid peroxidation and protein oxidation levels, and (iii) the relative expression of corresponding antioxidant genes (SOD, CAT, and GPx) in the Pacific oyster, one of the most commercially significant bivalve mollusks.

## 2. Materials and Methods

### 2.1. Animal Collection and Maintenance

Adult Pacific oysters *M. gigas* (5 years old, 74.3 ± 5.1 g, 12.2 ± 2.3 cm, n = 280) were sourced from Mariculture LLC, an aquaculture facility near Sevastopol (44.616014, 33.502248). Upon arrival at the laboratory, they were acclimatized for 7 days under controlled conditions [23]. Throughout acclimation and experimentation, the oysters were kept in 50–70 L aquariums at 18–20 °C, pH 8.2, dissolved oxygen 7–8 mg/L, and salinity 17–18‰. To eliminate metabolic wastes, two-thirds of the water volume was renewed daily. They were fed daily with the microalga *Tetraselmis viridis* (Rouchijajnen) R.E. Norris, Hori & Chihara, 1980, strain IBSS-25, supplied by the Department of Biotechnology and Phytoresources of the FRC IBSS RAS, at a fixed ration of 10^9^ cells per individual per day. No mortality occurred during either the acclimation or the experimental periods.

### 2.2. Preparation and Characterization of Phycobiliprotein (PBP) Extract

The aqueous PBP extract was derived from biomass of *L. platensis* (strain IBSS-31), obtained from the Shared Research Facility “Collection of Hydrobionts of the World Ocean” at FRC IBSS RAS. The cyanobacterium was cultivated in Zarrouk’s medium in open raceway ponds inside greenhouses at the IBSS experimental microalgae production facility [24].

For pigment recovery, distilled water was added to the biomass, and cell disruption was achieved via two freeze–thaw cycles to maximize PBP release [25]. Extraction continued in cold distilled water (5 °C) for 24 h. The resulting extract was then clarified by centrifugation (Eppendorf 5430R, Hamburg, Germany) at 7000 rpm for 10 min and stored in darkness at −18 °C [26].

Spectrophotometric analysis (UV-2600i, Shimadzu, Kyoto, Japan) was performed across 400–800 nm to determine pigment concentrations. Absorbance was recorded at the maxima for C-phycocyanin (C-PC, 620 nm) and allophycocyanin (A-PC, 650 nm), with a correction at 750 nm for nonspecific scattering. Concentrations were calculated using the established formulas [26]:

C-PC = 0.166 × D_620_ − 0.091 × D_650_ (1)

A-PC = 0.159 × D_650_ − 0.041 × D_620_ (2)

where D denotes optical density at the indicated wavelength.

The extract exhibited a concentration of 4000–4500 µg/mL, corresponding to a yield of 10 g PBP per 100 g biomass. After further centrifugation at 7000× *g*, purity was evaluated via the D620/D280 ratio, which also reflected the C-PC-to-A-PC proportion. A ratio of 0.78 confirmed that the extract met quality criteria for food and aquaculture uses. The average relative composition was 86% C-PC and 14% A-PC.

For experimental use, extracts were freshly prepared and kept frozen, then thawed at 4 °C and protected from light immediately before addition to tanks, in order to minimize pigment degradation [27].

### 2.3. Experimental Design

**Figure 1:**
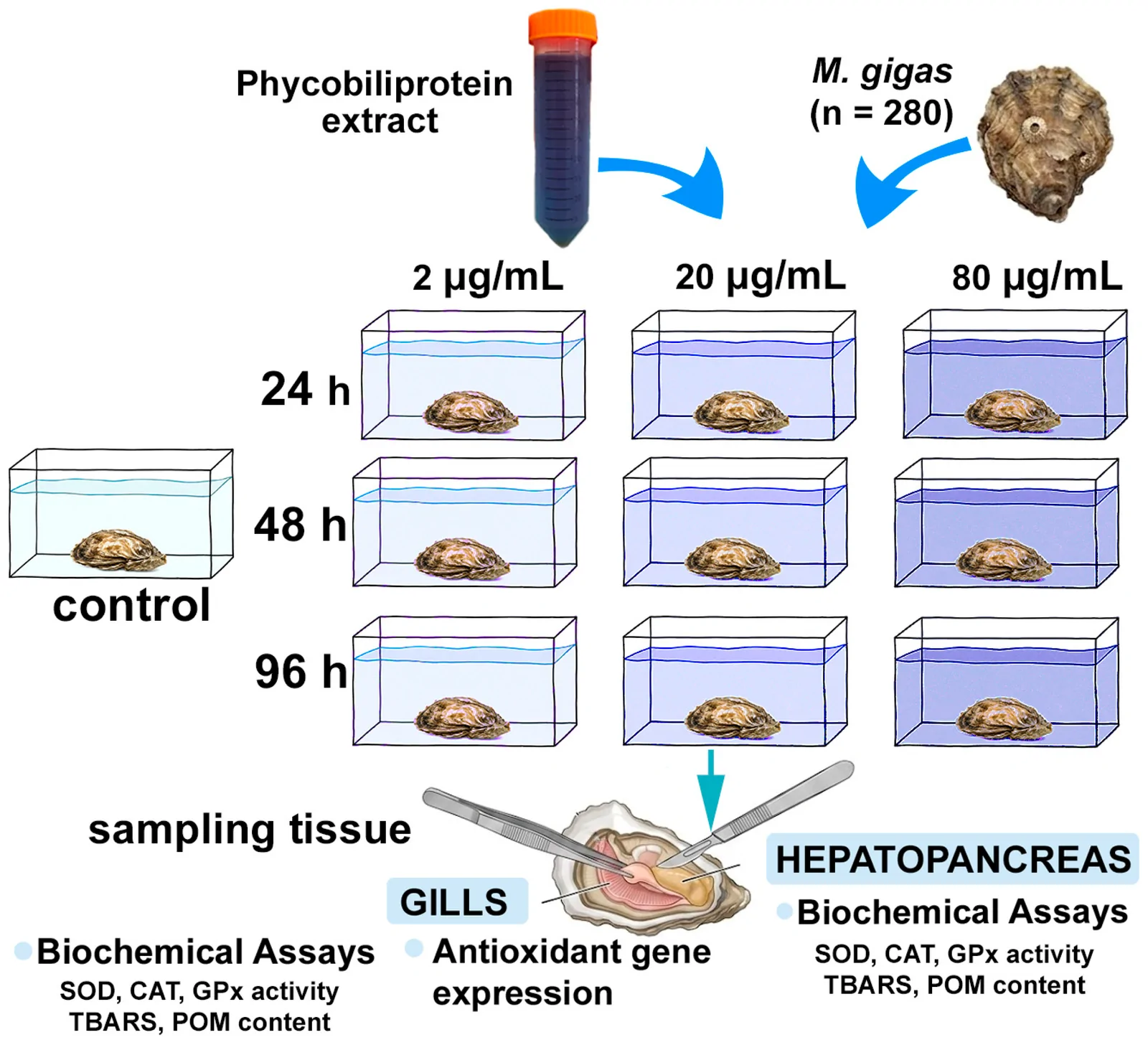
Schematic description of the experimental design.

Oysters were randomly distributed into four experimental groups: three treatment groups receiving different concentrations of PBP extract and one untreated control group. Each group was maintained in a separate tank containing 28 oysters per tank. The entire experiment, comprising all four tank treatments, was independently repeated three times (i.e., three independent experimental runs), yielding a total of 10 tanks across the three replicates. For statistical analysis, the tank was considered the true biological replicate for each treatment level. However, for biochemical and molecular assays, tissue samples were collected from multiple individual oysters within each tank to account for intra-tank biological variation. Specifically, at the end of the exposure period, 10 oysters were randomly selected from each tank for biochemical assays in gills and hepatopancreas, and for qPCR analysis. Throughout the exposure period, feeding was not suspended. The aqueous PBP extract was introduced into treatment tanks to reach final concentrations of 2, 20, and 80 μg/mL. These levels were chosen based on prior work reporting no toxic effects from the extract [28]. Control tanks received no extract. While the test compound was administered via waterborne exposure (aqueous extract added to tank water), this route serves as an effective proxy for dietary supplementation in filter-feeding bivalve mollusks. Given their continuous filtration activity, suspended compounds are ingested along with water and particulate matter, closely mimicking the digestive and absorptive processes associated with food-borne intake. Therefore, throughout this manuscript, we compare this exposure route with “dietary supplementation” in the context of filter-feeder physiology, with the understanding that the compound reaches the digestive tract via filtration rather than direct feed incorporation. Exposure lasted for 24, 48 and 96 h, during which a regular water exchange regimen (two-thirds volume replaced daily) was maintained, with corresponding extract replenishment to stabilize concentrations (Figure 1).

At the end of each incubation period, samples were collected from each group: gills and hepatopancreas for AO enzyme assays, lipid peroxidation and protein oxidative modification (n = 10), and gills for real-time PCR analysis (n = 10). All tissue samples for biochemical assays were dissected and immediately flash-frozen at −80 °C for later analysis.

### 2.4. Enzymatic Activity Assay

Frozen tissue samples were thawed on an ice bath and homogenized with a homogenizer Stegler S10 (DG-120) in a cold Tris/HCl buffer (20 mM, pH 7.5) containing 0.5 mM of ethylenediaminetetraacetic acid (EDTA). The homogenates were centrifuged at 11,000× *g* for 20 min at 4 °C to obtain supernatants [16], which were then kept on ice (0–4 °C) and immediately used for enzyme activity determinations. All enzymatic assays were performed in triplicate at 25.0 ± 0.5 °C [29]. Total protein content in the tissue samples was estimated according to the Lowry method [30].

GPx activity was quantified by measuring the accumulation of oxidized glutathione by Terziev et al. [31]. SOD activity was assessed based on the degree of inhibition of nitroblue tetrazolium reduction [32], while CAT activity was determined using a method that relies on the reaction of residual hydrogen peroxide with ammonium molybdate [33].

### 2.5. Oxidative Stress Parameters

Lipid peroxidation (LPO) levels in oyster tissues were estimated by measuring thiobarbituric acid-reactive substances (TBARS) according to the method of Ohkawa et al. [34], and results were expressed as relative units per mg of wet tissue weight.

The extent of protein oxidative modification (POM) was evaluated in both gill and hepatopancreas tissues using a procedure modified for marine organisms by Sigacheva et al. [35]. The assay is based on spectrophotometric detection of 2,4-dinitrophenylhydrazones of both neutral and basic character in the supernatant. Protein precipitation was achieved by adding 20% trichloroacetic acid to the supernatant, and the resulting pellet was redissolved for subsequent measurement of oxidative modifications. Neutral aldehyde and ketone derivatives were identified at 356 nm and 370 nm, while basic aldehyde and ketone products were recorded at 430 nm and 530 nm. The results were expressed as optical units per mg of protein in the sample.

### 2.6. RNA Extraction and Antioxidant Gene Expression

Total RNA was extracted from gill samples using ExtractRNA reagent (Evrogen, Moscow, Russia). There were 10 biological replicates for each group (control and experimental). RNA purity and integrity were assessed using the A260/A280 absorbance ratio with a Nano-Photometer™ N60 (GmBH Implen, Munich, Germany) and by electrophoresis on a 1.5% agarose gel stained with ethidium bromide, respectively. First-strand cDNA was synthesized from 500 ng of purified total RNA using MMLV Reverse Transcriptase (Evrogen, Moscow, Russia) following the manufacturer’s protocol.

Quantitative real-time PCR (qPCR) was performed on a LightCycler^®^ 96 Instrument (Roche, Basel, Switzerland) using a qPCRmix-HS SYBR Green I kit (Evrogen, Moscow, Russia). The reaction mixture (total volume 15 μL) contained 1 μL of cDNA template and 0.4 μM of each gene-specific primer. The thermal cycling conditions consisted of an initial denaturation at 95 °C for 3 min, followed by 40 cycles of 95 °C for 15 s, 60 °C for 15 s, and 72 °C for 15 s. Melting curve analysis was performed from 65 to 95 °C (increment of 0.5 °C/s to verify amplification specificity). A no-template control (NTC) was included for each primer set in every run.

In this study, the expression levels of the antioxidant genes CAT, Cu/Zn-SOD, and Mn-SOD were analyzed. Elongation factor 1-alpha (EF1α) was used as a reference gene. All reactions were performed in technical triplicate. Amplification efficiencies were determined using a standard curve generated from serial dilutions of cDNA. All gene-specific primer sequences are provided in Table 1.

Data analysis was carried out using LightCycler^®^ 96 software (v 1.1) (Mannheim, Germany). For each measurement, the quantification cycle (Cq) was determined as the fractional cycle number at which the fluorescence crosses the fixed threshold. The stability of the reference gene was evaluated using BestKeeper software v. 1 [36]. Relative gene expression was calculated using the 2^−ΔΔCq^ method [37], normalized to the EF1α reference gene.

### 2.7. Statistics

Statistical analysis and data visualization were performed using GraphPad Prism 9.5.0 software (GraphPad Software, San Diego, CA, USA). Normality of data distribution was assessed using the Kolmogorov–Smirnov and Shapiro–Wilk tests, while homogeneity of variances was evaluated with Levene’s test. Data that did not follow a normal distribution were normalized using the quantile normalization method. A two-way analysis of variance (ANOVA) was employed to evaluate the effects of time and extract concentration, with Dunnett’s test used for post hoc comparisons between groups. Differences were considered statistically significant at *p* < 0.05. Results are presented as mean ± standard error of the mean (SEM).

## 3. Results

**Figure 2:**
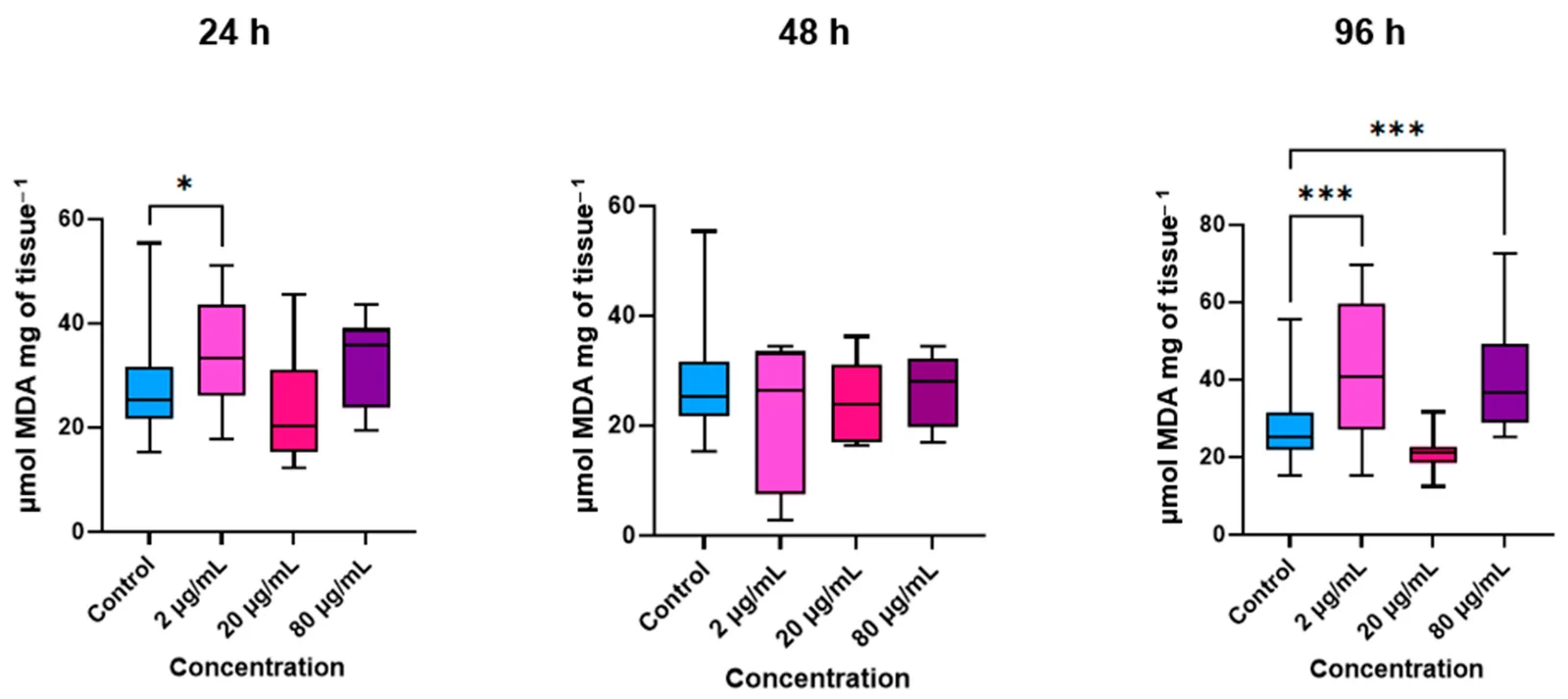
LPO levels in the gills of *M. gigas* exposed to PBPs (2, 20, and 80 µg/mL) for 24, 48, and 96 h. Data are expressed as μmol MDA mg^−1^ of tissue^−1^ and shown as mean ± SD (n = 10). Two-way ANOVA with Dunnett’s test: *—*p* ≤ 0.05, ***—*p* ≤ 0.001 vs. control.

In the gills of *M. gigas*, LPO levels, measured as TBARS, remained within the control range across most experimental groups. At the lowest tested concentration of the extract (2 µg/mL), LPO levels exhibited a fluctuating pattern: a significant increase was observed during the initial 24 h of exposure, followed by a return to baseline values after 48 h, and a subsequent rise again by the end of the 96 h incubation period (Figure 2). At higher extract concentrations, elevations in gill LPO were only evident after 96 h of exposure, and exclusively at the highest concentration tested (80 µg/mL).

**Figure 3:**
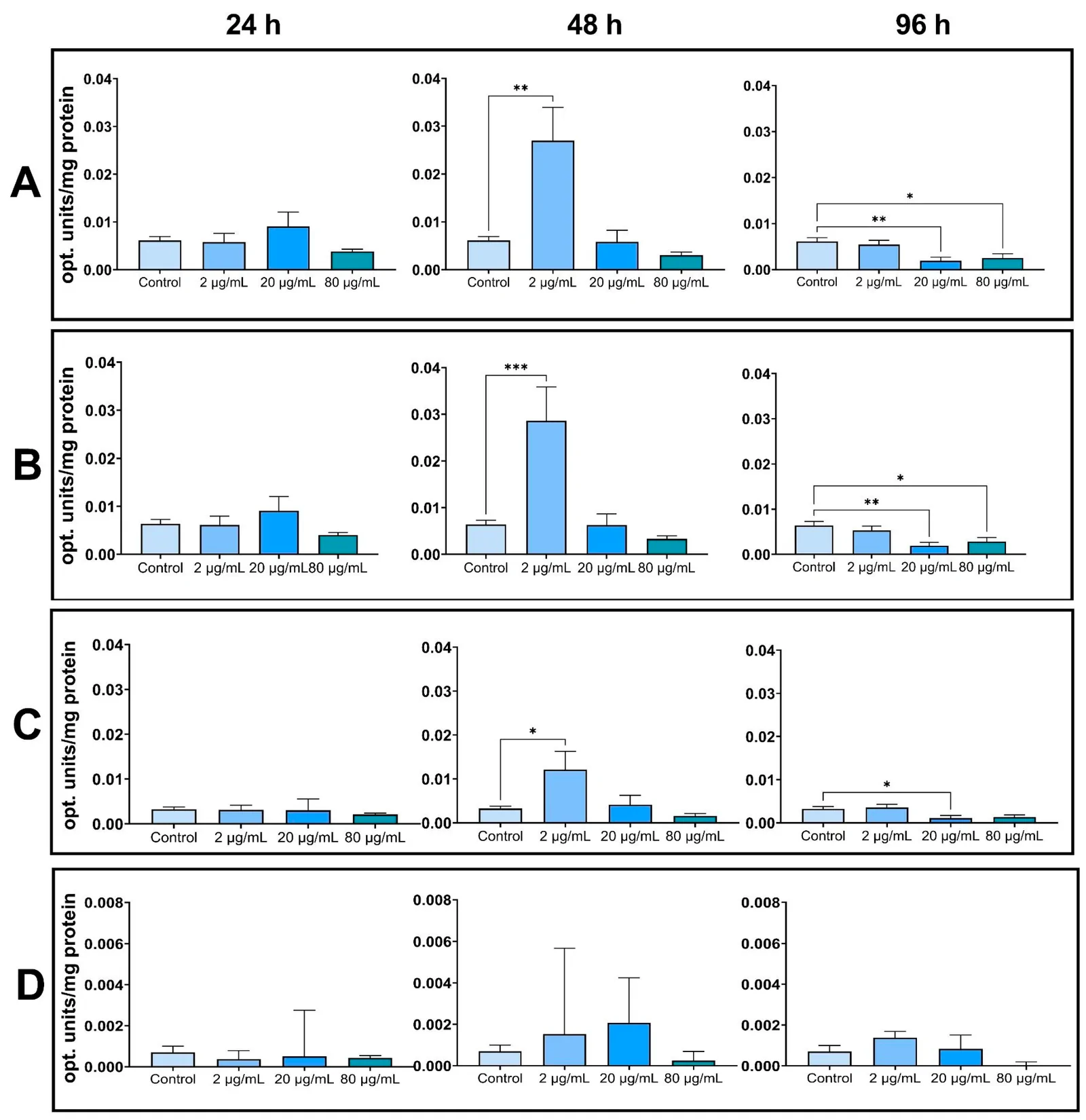
Content of neutral aliphatic aldehyde (**A**) and ketone (**B**) oxidatively modified protein (POM) derivatives, as well as alkaline aliphatic aldehyde (**C**) and ketone (**D**) POM derivatives in the gills of the *M. gigas* after 24, 48, and 96 h of exposure to aqueous extract of PBPs at concentrations of 2 µg/mL, 20 µg/mL, and 80 µg/mL. Values are expressed as optical units per mg protein (opt. units/mg protein) and shown as mean ± SD (n = 10). Two-way ANOVA followed by Dunnett’s multiple range test: *—*p* ≤ 0.05, **—*p* ≤ 0.01, ***—*p* ≤ 0.001 vs. control.

Treatment with the PBP extract resulted in an overall reduction in protein oxidative modification levels. This effect exhibited a marked concentration- and time-dependent pattern (Figure 3). Specifically, levels of various oxidized protein fractions—including aldehyde and ketone derivatives of both neutral and basic amino acid residues—were significantly decreased at the end of the exposure period in groups treated with the extract at 20 and 80 µg/mL. In contrast, at the lowest concentration tested (2 µg/mL), a transient increase in certain oxidized protein fractions was observed at the midpoint of the experimental period (48 h); however, these values had returned to control levels by the end of the 96 h incubation.

**Figure 4:**
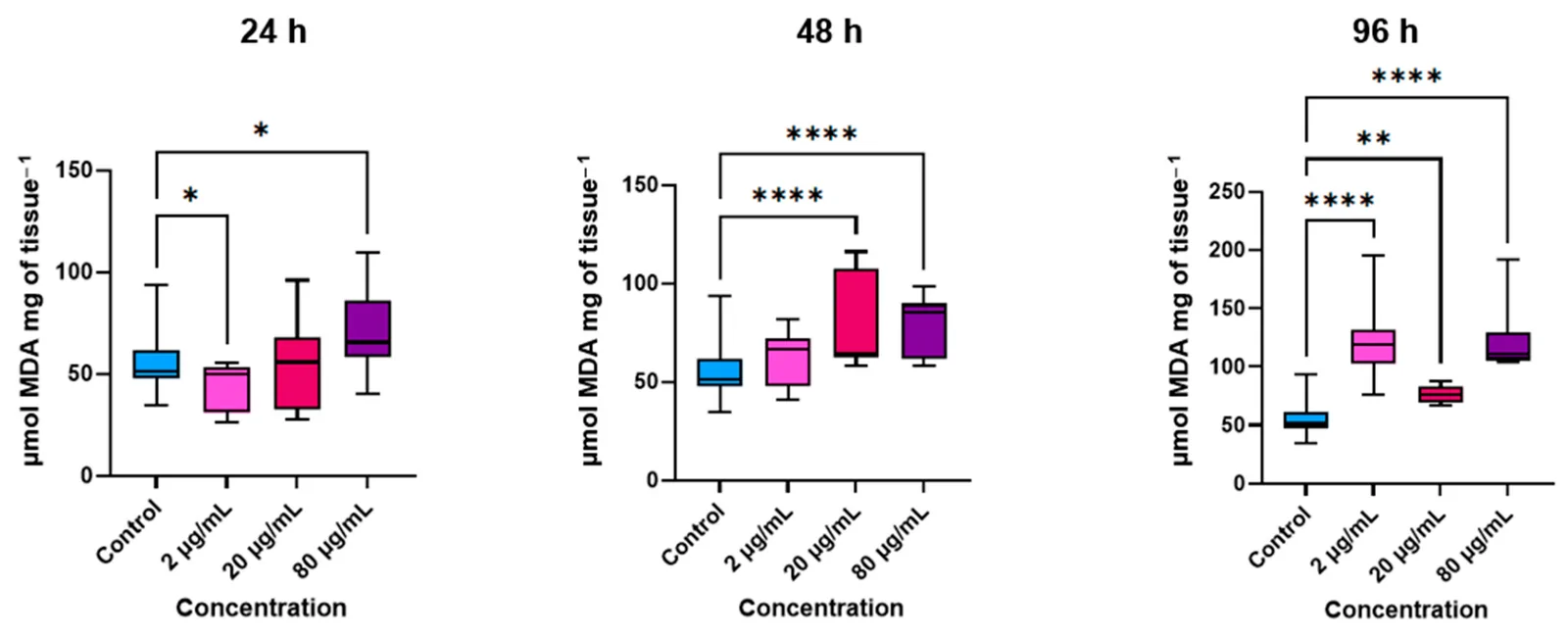
LPO levels in hepatopancreas of *M. gigas* exposed to PBPs (2, 20, and 80 µg/mL) for 24, 48, and 96 h. Data are expressed as μmol MDA mg^−1^ of tissue^−1^ and shown as mean ± SD (n = 10). Two-way ANOVA with Dunnett’s test: *—*p* ≤ 0.05, **—*p* ≤ 0.01, ****—*p* ≤ 0.0001 vs. control.

In the oyster hepatopancreas, application of the PBP extract elicited a more pronounced response, with a clear increase in LPO levels. Specifically, TBARS levels gradually rose in groups treated with 20 and 80 µg/mL of the extract starting from 48 h of exposure, and by 96 h, a significant elevation was also observed at the lowest tested concentration (2 µg/mL) (Figure 4).

**Figure 5:**
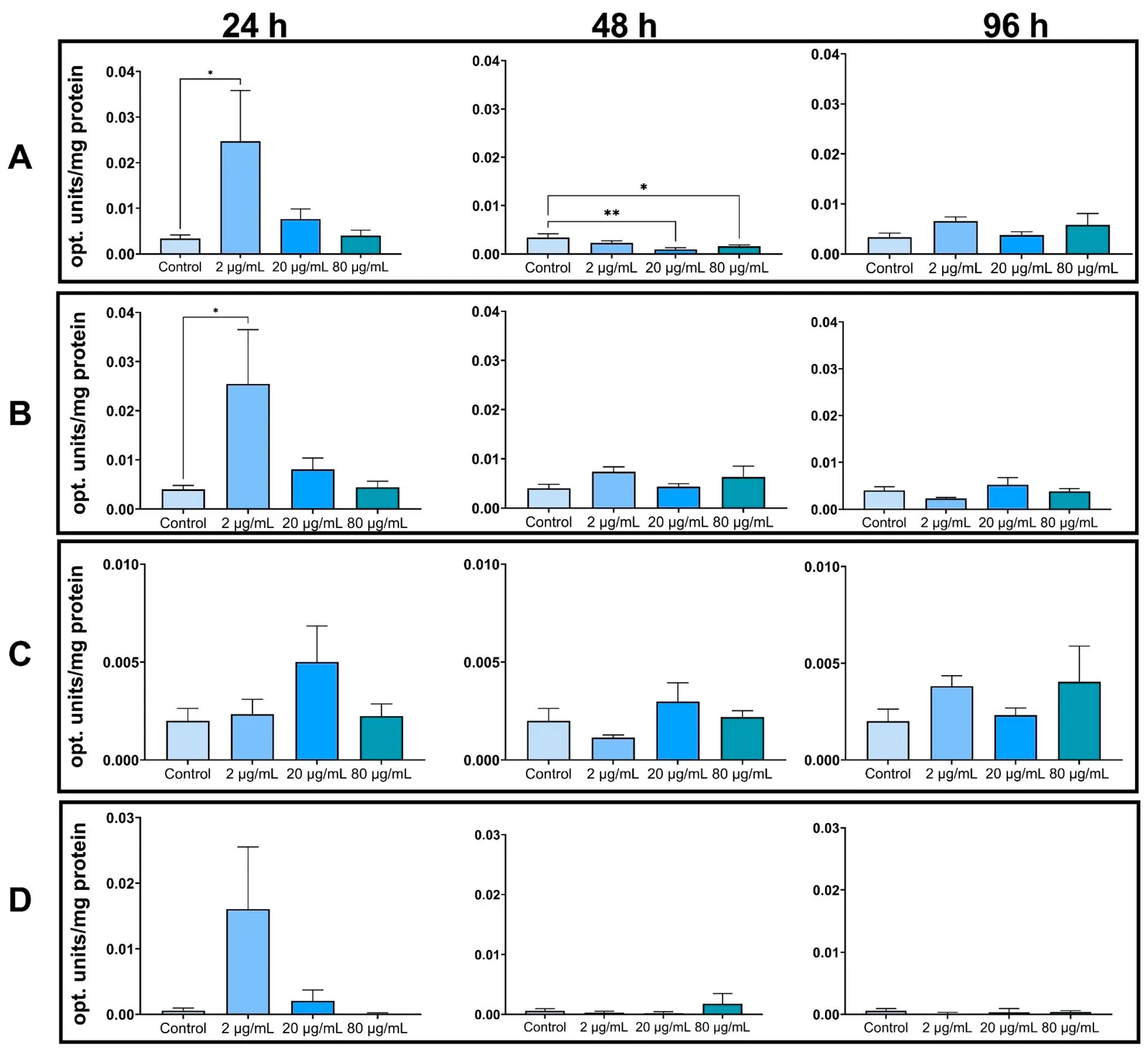
Content of neutral aliphatic aldehyde ((**A**), 356 nm) and ketone ((**B**), 370 nm) oxidatively modified protein (POM) derivatives, as well as alkaline aliphatic aldehyde ((**C**), 430 nm) and ketone ((**D**), 530 nm) POM derivatives in hepatopancreas of the *M. gigas* after 24, 48, and 96 h of exposure to aqueous extract of PBPs at concentrations of 2 µg/mL, 20 µg/mL, and 80 µg/mL. Values are expressed as optical units per mg protein (opt. units/mg protein) and shown as mean ± SD (n = 10). Two-way ANOVA followed by Dunnett’s multiple range test: *—*p* ≤ 0.05, **—*p* ≤ 0.01 vs. control.

Notably, exposure to the PBP extract did not induce protein oxidation in the oyster hepatopancreas across most experimental groups (Figure 5). A transient increase in neutral aldehyde and ketone derivatives was observed only during the first 24 h of exposure at the lowest concentration tested (2 µg/mL), after which levels normalized and remained within control values for the remainder of the observation period.

**Figure 6:**
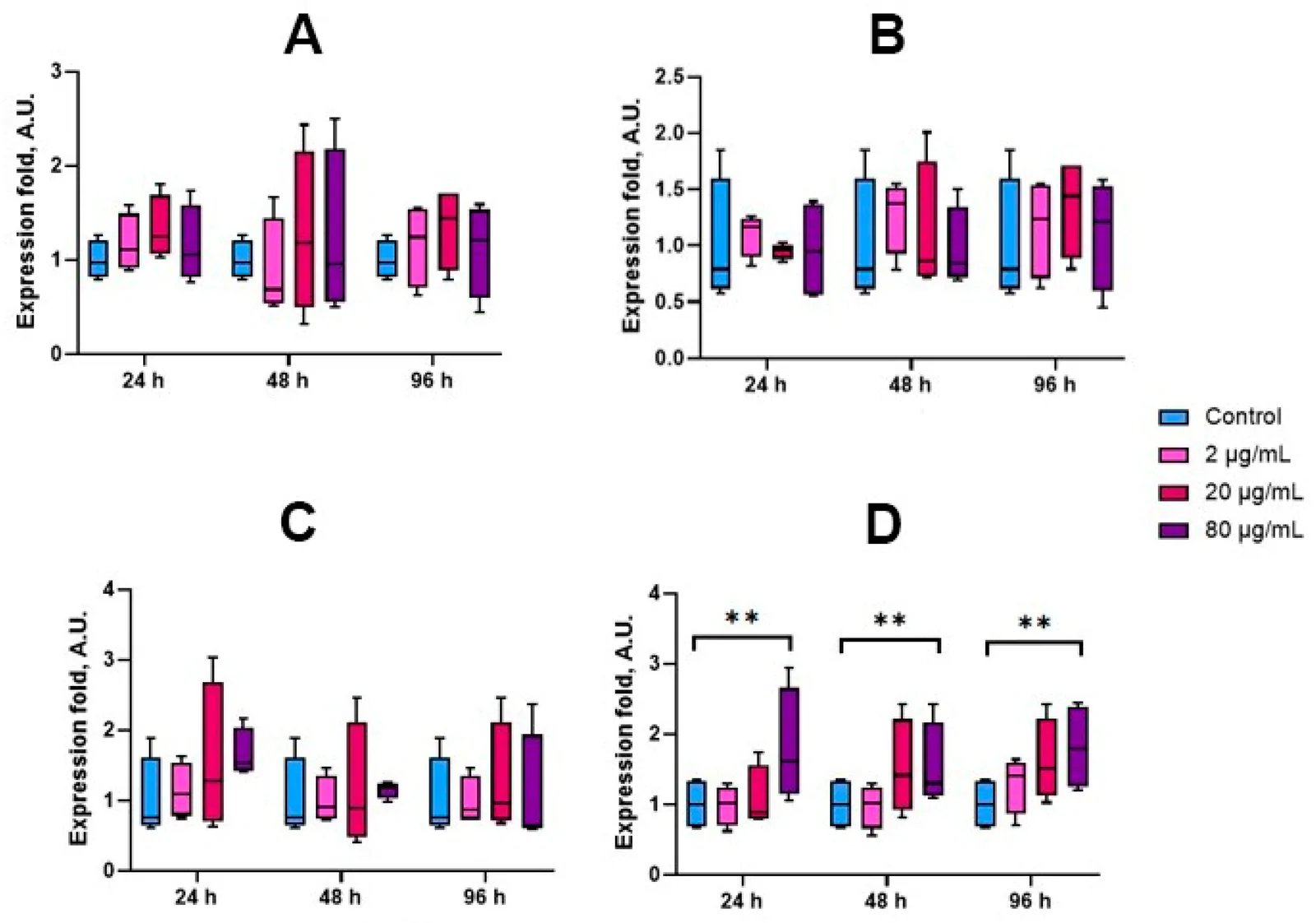
Relative mRNA expression levels of antioxidant enzyme genes in the gills of the *M. gigas* fed with graded levels of aqueous extract of PBPs—2 µg/mL, 20 µg/mL, and 80 µg/mL over 24 h, 48 h and 96 h: (**A**) expression of CAT; (**B**) expression of Mn-SOD; (**C**) expression of Cu-Zn-SOD; (**D**) expression of GPx. Values are expressed as mean ± SD (n = 10). Two-way ANOVA followed by Dunnett’s multiple range test: **—*p* ≤ 0.01 vs. control.

In the gills, the PBP extract did not significantly affect the transcriptional levels of key antioxidant enzyme genes throughout the exposure period, with the exception of GPx. Specifically, expression of the GPx gene was significantly upregulated in oysters treated with the highest concentration of the extract (80 µg/mL), whereas transcript levels of SOD and CAT remained unaltered across all treatment groups and time points (Figure 6).

**Figure 7:**
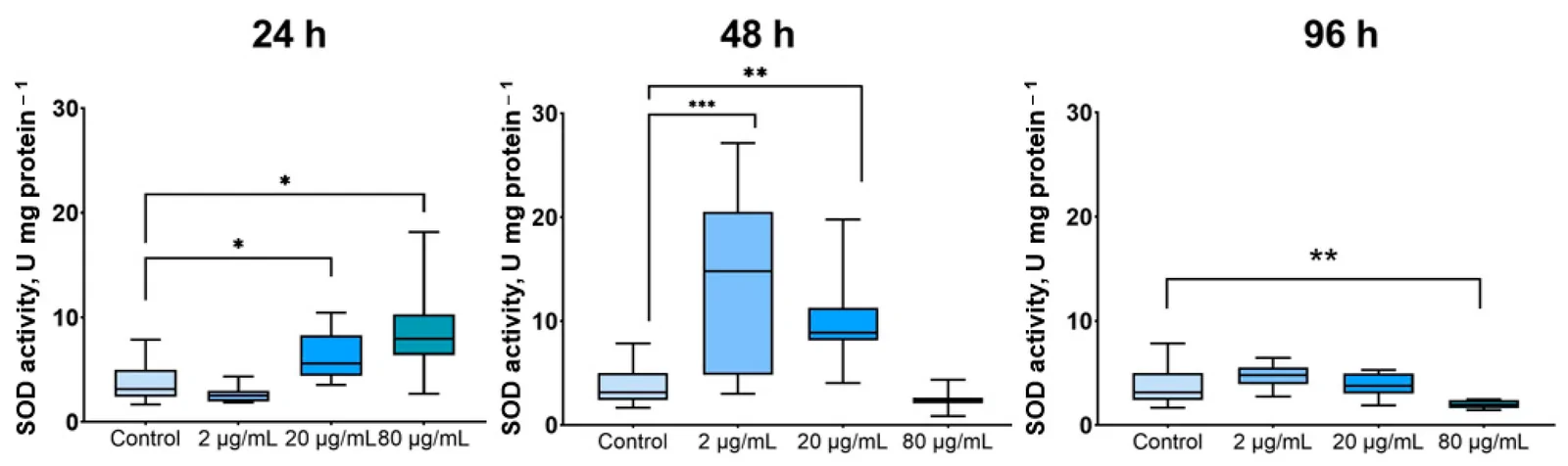
Activity of SOD in gills of the *M. gigas* fed with graded levels of aqueous extract of PBPs—2 µg/mL, 20 µg/mL, and 80 µg/mL over 24 h, 48 h and 96 h. Antioxidant enzyme activities were assessed spectrophotometrically. The differences between groups were assessed by two-way ANOVA followed by Dunnet multiple range test (*—*p* ≤ 0.05, **—*p* ≤ 0.01, ***—*p* ≤ 0.001, n = 10).

Exposure to the PBP extract induced changes in the activities of key antioxidant enzymes in oyster gills, with responses predominantly observed at low and intermediate concentrations. SOD activity was significantly elevated at 2 and 20 µg/mL after 48 h of exposure (*p* ≤ 0.01 to 0.001). A substantial increase in SOD activity was also recorded at 24 h in groups treated with 20 µg/mL and 80 µg/mL (*p* ≤ 0.05 for both). In contrast, a significant reduction in SOD activity was observed at the highest concentration (80 µg/mL) after 96 h of exposure (*p* ≤ 0.01) (Figure 7).

**Figure 8:**
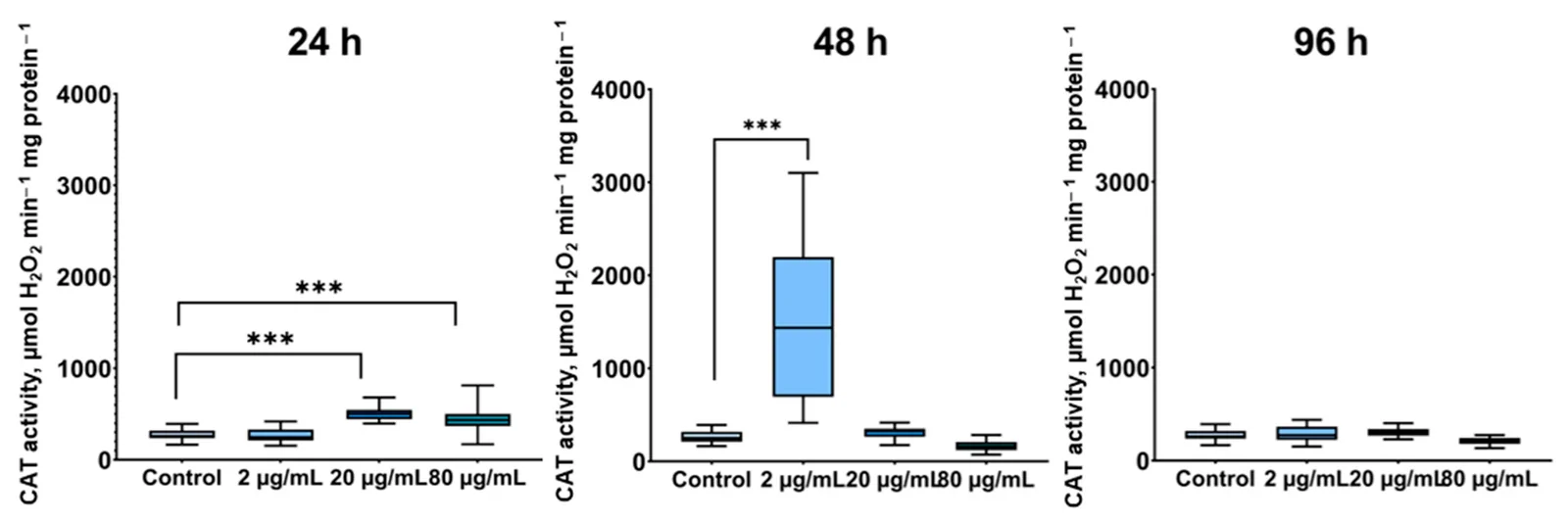
Activity of CAT in gills of the *M. gigas* fed with graded levels of aqueous extract of PBPs—2 µg/mL, 20 µg/mL, and 80 µg/mL over 24 h, 48 h and 96 h. Antioxidant enzyme activities were assessed spectrophotometrically. The differences between groups were assessed by two-way ANOVA followed by Dunnet multiple range test (***—*p* ≤ 0.001, n = 10).

CAT activity exhibited a similar pattern, increasing primarily at low and intermediate PBP concentrations: significant elevations were noted in the 2 µg/mL group at 48 h (*p* ≤ 0.001) and in the 20 µg/mL group at 24 h (*p* ≤ 0.001), as well as in the 80 µg/mL group at 24 h (*p* ≤ 0.001) (Figure 8). No statistically significant changes were detected in the remaining experimental groups.

**Figure 9:**
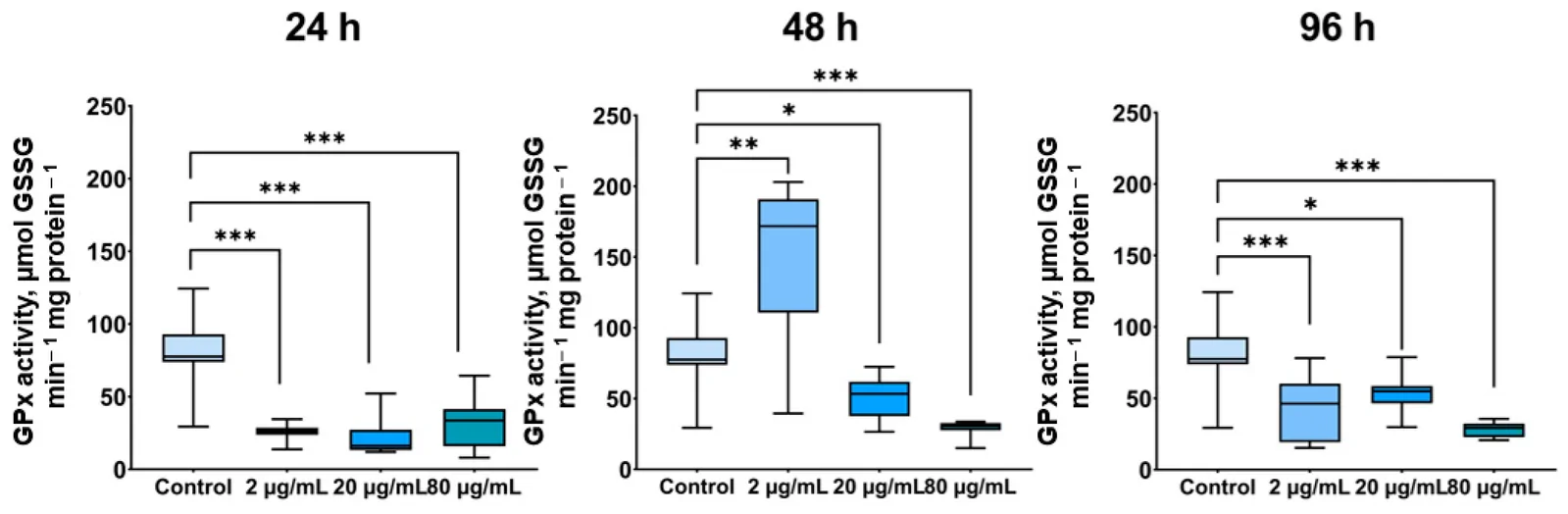
Activity of GPx in gills of the *M. gigas* fed with graded levels of aqueous extract of PBPs—2 µg/mL, 20 µg/mL, and 80 µg/mL over 24 h, 48 h and 96 h. Antioxidant enzyme activities were assessed spectrophotometrically. The differences between groups were assessed by two-way ANOVA followed by Dunnet multiple range test (*—*p* ≤ 0.05, **—*p* ≤ 0.01, ***—*p* ≤ 0.001, n = 10).

GPx activity showed a more complex response. A significant increase was observed only in the 2 µg/mL group at 48 h (*p* ≤ 0.01). However, GPx activity was markedly reduced in several other groups, predominantly at the highest extract concentration (80 µg/mL), across all exposure time points (*p* ≤ 0.01 to 0.001) (Figure 9).

**Figure 10:**
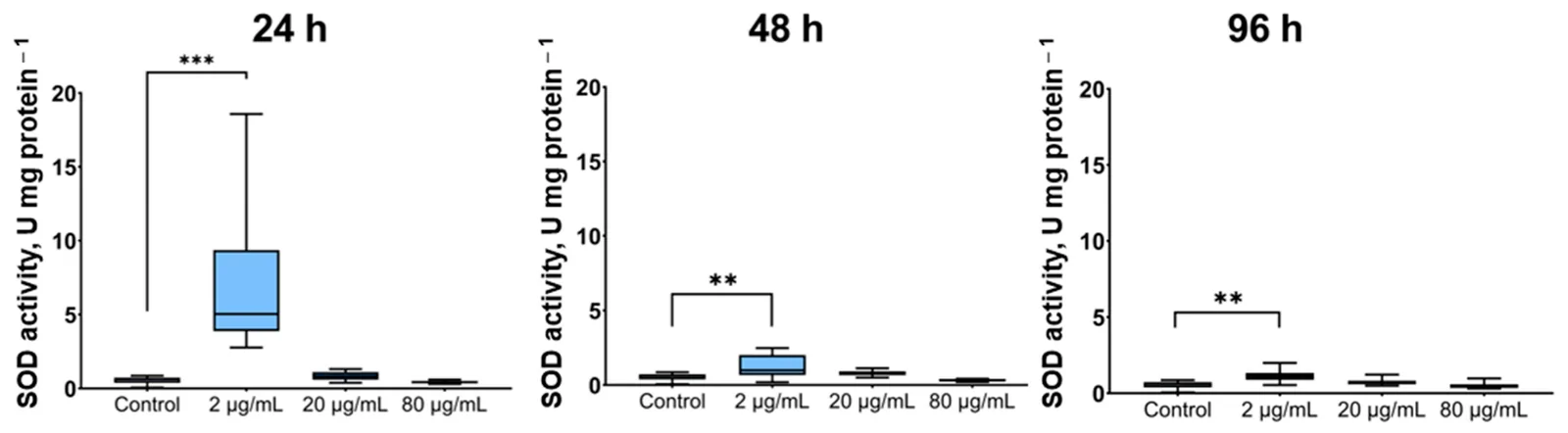
Activity of SOD in hepatopancreas of the *M. gigas* fed with graded levels of aqueous extract of PBPs—2 µg/mL, 20 µg/mL, and 80 µg/mL over 24 h, 48 h and 96 h. Antioxidant enzyme activities were assessed spectrophotometrically. The differences between groups were assessed by two-way ANOVA followed by Dunnet multiple range test (**—*p* ≤ 0.01, ***—*p* ≤ 0.001, n = 10).

As observed in the gills, the most pronounced increases in antioxidant enzyme activities in the oyster hepatopancreas occurred at low PBP concentrations. SOD activity was significantly elevated in groups treated with 2 µg/mL after 24 h, 48 h and 96 h of exposure (*p* ≤ 0.01 to 0.001) (Figure 10).

**Figure 11:**
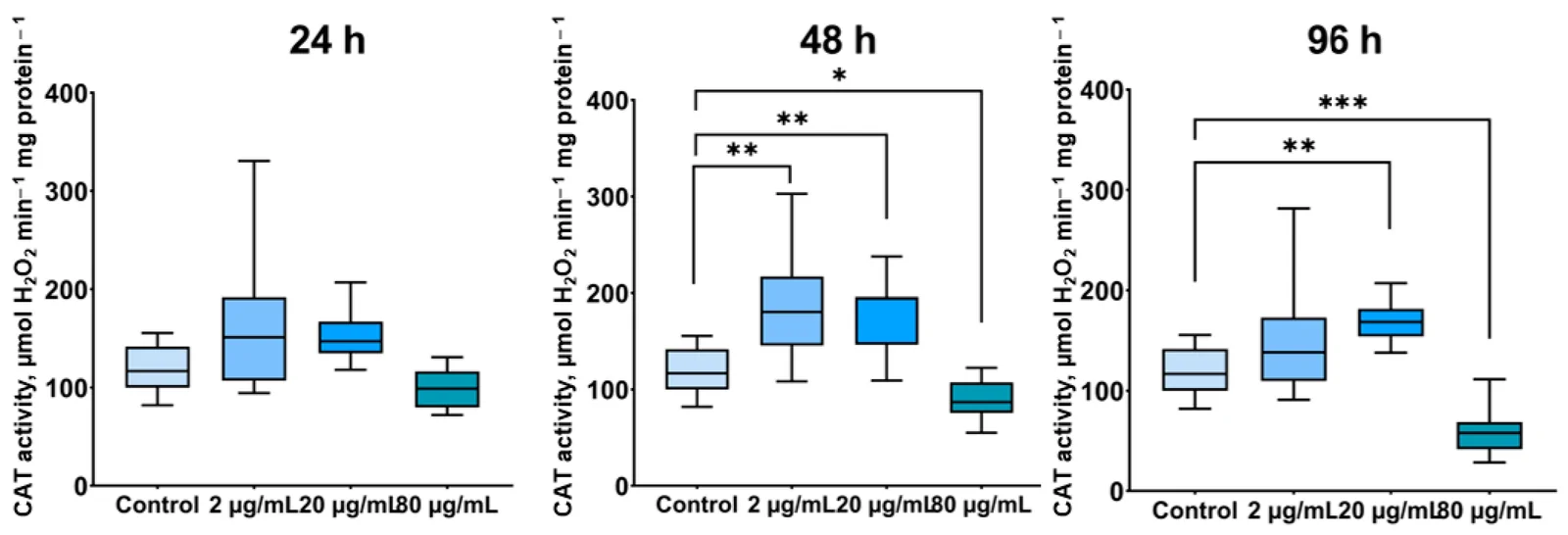
Activity of CAT in hepatopancreas of the *M. gigas* fed with graded levels of aqueous extract of PBPs—2 µg/mL, 20 µg/mL, and 80 µg/mL over 24 h, 48 h and 96 h. Antioxidant enzyme activities were assessed spectrophotometrically. The differences between groups were assessed by two-way ANOVA followed by Dunnet multiple range test (*—*p* ≤ 0.05, **—*p* ≤ 0.01, ***—*p* ≤ 0.001, n = 10).

CAT activity in the oyster hepatopancreas increased at 2 µg/mL and 20 µg/mL after 48 h (*p* ≤ 0.01 for both), as well as at 20 µg/mL following 96 h of exposure (*p* ≤ 0.001). In contrast, CAT activity was significantly decreased in groups treated with 80 µg/mL at both 48 h (*p* ≤ 0.05) and 96 h (*p* ≤ 0.001) (Figure 11).

**Figure 12:**
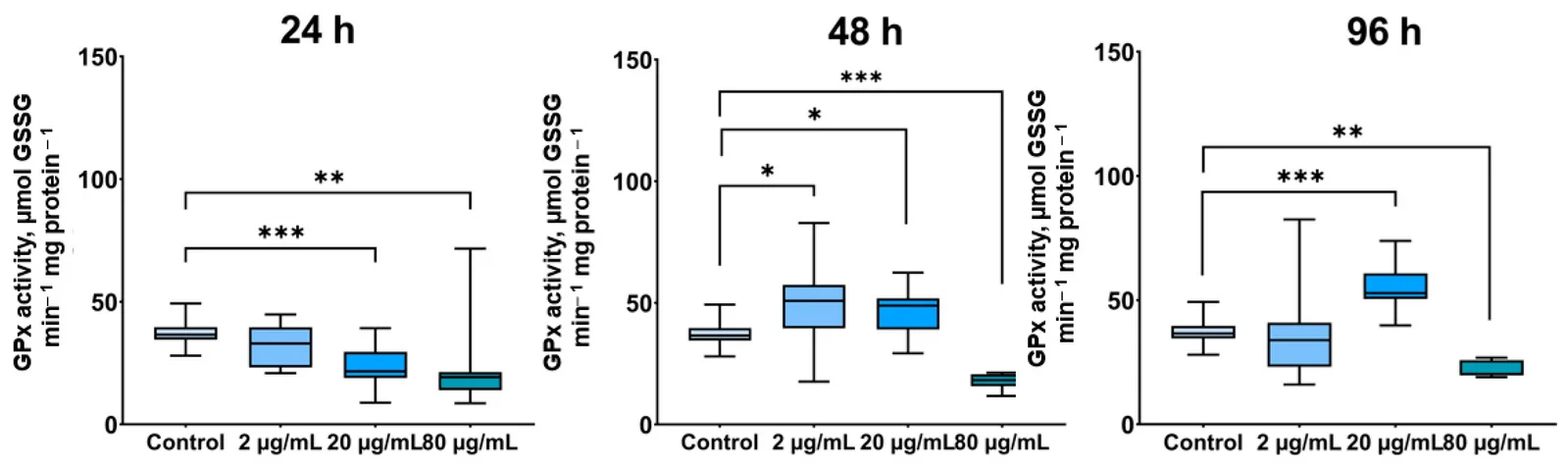
Activity of GPx in hepatopancreas of the *M. gigas* fed with graded levels of aqueous extract of PBPs—2 µg/mL, 20 µg/mL, and 80 µg/mL over 24 h, 48 h and 96 h. Antioxidant enzyme activities were assessed spectrophotometrically. The differences between groups were assessed by two-way ANOVA followed by Dunnet multiple range test (*—*p* ≤ 0.05, **—*p* ≤ 0.01, ***—*p* ≤ 0.001, n = 10).

GPx activity, similar to the pattern observed in the gills, was substantially reduced predominantly at the highest PBP concentration across all time points (*p* ≤ 0.01–0.001), as well as in the 20 µg/mL group at 24 h (*p* ≤ 0.001). Increases in GPx activity were only detected in the 2 µg/mL and 20 µg/mL groups at 48 h (*p* ≤ 0.05 for both), and in the 20 µg/mL group at 96 h (*p* ≤ 0.001) (Figure 12).

## 4. Discussion

*L. platensis* is a multicellular filamentous cyanobacterium in which C-PCs can constitute up to 40% of the total protein content [38]. These protein-pigment complexes serve as major light-harvesting antennae and are recognized for both their nutritional and medicinal properties [38]. Consistent with this, numerous studies have reported that PBPs exhibit antioxidant and anti-inflammatory activities across a wide range of vertebrate and invertebrate species, including aquatic organisms [38]. Furthermore, PBPs share structural similarities with bilirubins, and this homology has been proposed to underlie their antioxidant capacity [39,40]. Despite this growing body of evidence, the potential contribution of PBPs to the antioxidant defense system of commercially important bivalves remains largely unexplored. In this context, recent studies have highlighted the combined effects of environmental stressors on bivalve immunity, including the interactive effects of seawater acidification and high temperature on hemocyte parameters in *Mytilus coruscus* [41], as well as the antioxidant response of *Crassostrea hongkongensis* exposed to diel-cycling hypoxia under different salinities [42], underscoring the need to better understand oxidative stress modulation in commercially important bivalves.

The present study was designed to address this knowledge gap by evaluating the effects of a PBP extract on antioxidant defense parameters in *M. gigas* over a 96 h exposure period. The results demonstrate that PBP administration elicited a pronounced, dose- and time-dependent response in both gills and hepatopancreas, confirming the substantial biological activity of the tested extract.

The most notable finding of this study was the biphasic nature of the antioxidant enzyme response. Specifically, the most pronounced stimulatory effects on SOD and CAT activities occurred at low and intermediate concentrations (2 and 20 µg/mL) following 24 and 48 h of exposure, with all three enzymes exhibiting increased activity in the 2 µg/mL group at 48 h, except for SOD in the hepatopancreas. This pattern suggests that, at these concentrations, PBPs exert a direct antioxidant effect, presumably through the scavenging of ROS and subsequent upregulation of enzymatic antioxidant defenses.

This interpretation is supported by previous reports demonstrating that PBPs can directly neutralize ROS and enhance antioxidant enzyme activities in aquatic organisms. For instance, dietary PBP supplementation in *Mugil liza* resulted in elevated SOD, CAT, and total antioxidant capacity in tissues, accompanied by reduced MDA levels [43]. Similarly, total antioxidant activity was enhanced in the liver of *Mugil liza* fed a PBP-supplemented diet [44]. Consistent with our results, increased SOD and CAT activities were observed in the gills of *Oreochromis niloticus* following dietary administration of PBPs derived from *L. platensis* [19,45,46,47]. Comparable effects have also been documented in crustaceans, including *L. vannamei* [48].

In contrast, the highest concentration tested (80 µg/mL) consistently suppressed enzyme activities, particularly after prolonged exposure (48–96 h). This inhibitory effect manifested as decreased SOD activity at 96 h in the gills; reduced CAT activity at 48 and 96 h in the hepatopancreas; and, most notably, suppressed GPx activity across the most groups, except for the “2 μg/mL, 48 h” in the gills and “2 μg/mL, 24 h”, “20 μg/mL, 48 h”, and “20 μg/mL, 96 h” groups in the hepatopancreas. These findings indicate that the 80 µg/mL concentration may exceed the optimal range for stimulating antioxidant defenses, potentially inducing oxidative stress rather than mitigating it.

A striking observation was the differential sensitivity of the two tissues examined. The hepatopancreas exhibited more pronounced and frequent elevations in LPO levels compared with the gills—across all exposure periods at 20 and 80 µg/mL—with the exception of the 20 µg/mL group at 24 h. This tissue-specific difference may be attributable to the distinct physiological role of the hepatopancreas as the primary organ for detoxification and biotransformation of xenobiotics and other exogenous compounds [49,50]. Dietary administration of PBP extracts may impose an additional oxidative burden on the digestive system due to the presence of ballast proteins and fibrous components in the extract. These substances may be difficult to digest and could promote the formation of incomplete digestion products [51,52], thereby exacerbating LPO processes. Consistent with this interpretation, efficient digestion of fibrous material and maximal protein assimilation from algal sources may require considerable metabolic resources [51]. This likely explains why the increase in LPO levels and the suppression of antioxidant activity were more pronounced in the hepatopancreas than in the gills of the examined oysters.

In contrast to the LPO data, the effects on protein oxidation were predominantly protective. Elevated levels of protein oxidative modification products were observed only transiently at early stages of PBP exposure—specifically, in the 2 µg/mL group at 48 h in the gills and in the 2 µg/mL group at 24 h in the hepatopancreas. However, by the end of the 96 h exposure period, a concurrent reduction in protein oxidative modification products was detected in both tissues at 20 and 80 µg/mL, suggesting a net protective effect of PBPs against protein oxidation. This finding is noteworthy because it indicates that even concentrations that suppressed enzyme activity (80 µg/mL) ultimately reduced protein damage, implying that PBPs may exert protein-protective effects independent of their influence on endogenous antioxidant enzymes. As noted above, this is likely attributable to the potent antioxidant and protein-protective properties of these pigments, which are associated with their abundance of non-polar and sulfur-containing amino acids that enhance structural stability and bioactivity—not only of the algal proteins themselves but also of proteins in consumer organisms following dietary uptake [53].

A notable finding was the selective upregulation of GPx gene expression in the gills at 80 µg/mL, whereas SOD and CAT transcript levels remained unchanged. This observation is striking because it contrasts with the enzymatic activity data, where GPx activity was consistently suppressed at this concentration. This dissociation between transcript levels and enzymatic activity suggests that PBPs may influence GPx at multiple regulatory levels—stimulating transcription while simultaneously inhibiting enzyme function or stability, possibly through post-translational mechanisms or substrate limitation. Furthermore, the fact that only GPx, among the three genes examined, responded transcriptionally suggests that GPx may represent a responsive molecular target for PBP-mediated regulation in oyster gills.

However, information regarding the effects of spirulina extracts on GPx expression in aquatic and terrestrial animals remains limited. Tayag et al. demonstrated that SOD and GPx transcript levels, as well as enzymatic activities, increased in parallel with rising concentrations of *L. platensis* extract in the white shrimp *L. vannamei* [48]. Stimulatory effects of *L. platensis* extract on GPx activity have also been reported in *Oreochromis niloticus* [54]. In a different context, upregulation of GPx expression following *L. platensis* extract administration was associated with improved auditory function in the mouse cochlea [55]. Elevated GPx activity, along with increased glutathione-S-transferase activity and glutathione levels, was observed in tissues of grass carp (*Ctenopharyngodon idella*) fed a *L. platensis* extract-supplemented diet [55]. Additionally, PBPs have been shown to increase GPx activity in human pancreatic beta-cells [56]. Taken together, these findings indicate that GPx is a common target of PBP action across diverse taxa, although the direction and magnitude of the response appear to be context- and dose-dependent.

The suppression of antioxidant enzyme activities at 80 µg/mL warrants mechanistic consideration. The present study observed elevated LPO levels in the 2 µg/mL group at 96 h, as well as across all groups treated with 80 µg/mL, indicating enhanced ROS generation under these conditions. This oxidative stress, in turn, likely contributes to the observed suppression of antioxidant enzyme activities. Furthermore, GPx appeared to be particularly sensitive to these effects, as its activity was consistently reduced at 80 µg/mL across all exposure periods in both tissues.

An alternative explanation of these results involves the potential toxicity of spirulina extracts at elevated doses. Indeed, in vivo studies on *Danio rerio* larvae demonstrated that C-PC extracts were non-toxic at lower concentrations and provided complete protection against hydrogen peroxide-induced mortality; however, higher PBP concentrations exhibited overt toxic effects [57]. This toxicity may be related to the presence of residual ballast proteins in the extract, which could lead to excessive protein loading in oyster tissues. A similar pattern has been observed in studies on *M. gigas*, where high PBP doses (200 µg/L) resulted in decreased activity of nonspecific cytoplasmic esterases and increased DNA damage in hemocytes, indicative of genotoxic effects [28].

Importantly, the reduction in antioxidant enzyme activities following high-dose PBP exposure is not without precedent. Consistent with our findings, dietary inclusion of 2–10% *L. platensis* over eight weeks decreased SOD, CAT, and GPx activities in several fish species [58], presumably because elevated intake of fat-soluble antioxidants may reduce the physiological demand for endogenous enzymatic antioxidants [59,60]. Similarly, reduced antioxidant enzyme activities following *L. platensis* supplementation have been documented in common carp (*Cyprinus carpio*), Nile tilapia (*Oreochromis niloticus*), and goldfish (*Carassius auratus*) [43]. These observations suggest that *S. platensis* possesses potent singlet oxygen-quenching capacity and can act as a direct antioxidant to neutralize free radicals, thereby diminishing the need for upregulated endogenous enzymatic defenses [61,62]. This interpretation aligns with the hormesis concept, wherein moderate antioxidant supplementation enhances endogenous defenses, whereas excessive supplementation may suppress them due to reduced oxidative signaling [60].

## 5. Conclusions

In summary, the present findings indicate that PBP extract exerts dose-dependent effects that can be conceptualized within a biphasic framework. At low to intermediate concentrations (2–20 µg/mL), PBPs enhance endogenous antioxidant enzyme activities (SOD and CAT) and reduce protein oxidation, consistent with their known radical-scavenging properties. At high concentrations (80 µg/mL), however, PBPs suppress enzyme activities—particularly GPx—while simultaneously increasing LPO, suggesting that excessive dosing may overwhelm the oyster’s antioxidant capacity. Even at high concentrations, PBPs retain their protein-protective effects, indicating that their radical-scavenging activity may be partially uncoupled from their influence on enzyme expression. The selective upregulation of GPx transcription at 80 µg/mL further suggests that different PBPs may exert distinct regulatory effects at the transcriptional versus post-transcriptional level.

Taken together, these results indicate that PBPs derived from *L. platensis* represent a promising functional feed additive capable of modulating the oxidative status of *M. gigas*, although optimal dosage and exposure duration are critical determinants of their efficacy and safety. However, further studies are needed to evaluate the efficacy and safety of PBPs when administered as dietary supplements, as the mode of administration, bioavailability, and physiological responses may differ substantially from waterborne exposure. Optimal dosage and exposure duration remain critical factors.

## Figures and Tables

**Table 1:** Real-time quantitative PCR primers for antioxidant defense genes and the elongation factor 1-alpha (EF1α) reference gene of the *M. gigas*.

Gene Name	Forward 5′–3′	Reverse 5′–3′	GenBank Accession Number
Sod Mn	CAAAGTCAATCAGTGCCCT	CATTGCCTCTGCCAGT	U420128
Sod Cu/Zn	CAGAGGATCACGAGAGGC	CGTTTCCGGTCGTCTT	J496219
Cat	TCGTCATATCGGGTTTACTTCTG	CTTGTCACGTCCTGCCATT	M853618
EF1α	GTCACCAAGGCTGCACAGAAAG	CCGACGTATTTCTTTGCGATGT	B122066.1

## Data Availability

Data will be made available on request.

## Abbreviations

PBP	Phycobiliprotein
C-PC	C-phycocyanin
APC	Allophycocyanin
SOD	Superoxide dismutase
CAT	Catalase
MDA	Malondialdehyde
GPx	Glutathione peroxidase
FRC	Federal Research Center
IBSS	Institute of Biology of the Southern Seas
RAS	Russian Academy of Sciences
A-PC	Allophycocyanin
LPO	Lipid peroxidation
TBARS	Thiobarbituric acid-reactive substances
NTC	No-template control
EF1α	Elongation factor 1-alpha
Cq	Quantification cycle
SEM	Standard error of the mean
POM	Oxidatively modified protein
